# Tetra­amminelithium triamminelithium tris­ulfide, [Li(NH_3_)_4_][Li(NH_3_)_3_S_3_]

**DOI:** 10.1107/S1600536812043863

**Published:** 2012-10-27

**Authors:** Christian Guentner, Nikolaus Korber

**Affiliations:** a Institut für Anorganische Chemie, Universität Regensburg, Universitätsstrasse 31, 93053 Regensburg, Germany

## Abstract

The title compound, [Li(NH_3_)_4_]^+^[Li(NH_3_)_3_S_3_]^−^, an ammo­niate of the previously unknown lithium tris­ulfide, was obtained from the reaction of lithium and sulfur in liquid ammonia. The asymmetric unit consists of two crystallographically independent formula units. The [Li(NH_3_)_4_]^+^ cations are close to regular LiN_4_ tetra­hedra. The anions contain LiSN_3_ tetra­hedra; the S—S—S bond angles are 110.43 (5) and 109.53 (5)°. In the crystal, the components are linked by multiple N—H⋯S hydrogen bonds. A weak N—H⋯N hydrogen bond is also present.

## Related literature

For structural details of [Li(NH_3_)_4_]Se_3_, see: Brandl (2009 ▶). For N—H⋯S hydrogen bonds, see: Rossmeier (2002 ▶, 2005 ▶); Meier (2008 ▶). For the synthesis of tris­ulfides of the heavier alkali metals (Na–Cs), see: Böttcher (1977 ▶, 1980*a* ▶,*b* ▶). For hydrogen bonding, see: Steiner (2002 ▶).

## Experimental

### 

#### Crystal data

[Li(NH_3_)_4_][Li(NH_3_)_3_S_3_]
*M* *_r_* = 229.30
Monoclinic, 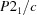

*a* = 12.422 (3) Å
*b* = 9.3721 (19) Å
*c* = 22.269 (5) Å
β = 92.46 (3)°
*V* = 2590.2 (9) Å^3^
*Z* = 8
Mo *K*α radiation
μ = 0.54 mm^−1^
*T* = 123 K
0.1 × 0.1 × 0.1 mm

#### Data collection

Stoe IPDS 1 diffractometer
Absorption correction: numerical (*X-SHAPE* and *X-RED*; Stoe & Cie 2006) ▶ *T*_min_ = 0.947, *T*_max_ = 0.981
32304 measured reflections
4788 independent reflections
2989 reflections with *I* > 2σ(*I*)
*R*_int_ = 0.097

#### Refinement

*R*[*F* ^2^ > 2σ(*F* ^2^)] = 0.040
*wR*(*F* ^2^) = 0.079
*S* = 0.81
4788 reflections
383 parameters
All H-atom parameters refined
Δρ_max_ = 0.53 e Å^−3^
Δρ_min_ = −0.26 e Å^−3^

### 

Data collection: *X-AREA* (Stoe & Cie, 2006 ▶); cell refinement: *X-AREA*; data reduction: *X-AREA*; program(s) used to solve structure: *SHELXS97* (Sheldrick, 2008 ▶); program(s) used to refine structure: *SHELXL97* (Sheldrick, 2008 ▶); molecular graphics: *DIAMOND* (Brandenburg, 2008 ▶); software used to prepare material for publication: *publCIF* (Westrip, 2010 ▶).

## Supplementary Material

Crystal structure: contains datablock(s) I, global. DOI: 10.1107/S1600536812043863/hb6966sup1.cif

Structure factors: contains datablock(s) I. DOI: 10.1107/S1600536812043863/hb6966Isup2.hkl

Additional supplementary materials:  crystallographic information; 3D view; checkCIF report

## Figures and Tables

**Table 1:** Selected bond lengths (Å)

Li1—N2	2.059 (7)
Li1—N3	2.061 (6)
Li1—N1	2.099 (7)
Li1—N4	2.104 (7)
Li2—N7	2.033 (7)
Li2—N5	2.066 (7)
Li2—N6	2.071 (7)
Li2—S1	2.547 (6)
Li3—N10	2.081 (6)
Li3—N11	2.082 (7)
Li3—N9	2.085 (7)
Li3—N8	2.105 (7)
Li4—N13	2.052 (7)
Li4—N14	2.058 (7)
Li4—N12	2.084 (7)
Li4—S4	2.503 (5)

**Table 2:** Hydrogen-bond geometry (Å, °)

*D*—H⋯*A*	*D*—H	H⋯*A*	*D*⋯*A*	*D*—H⋯*A*
N5—H5*A*⋯S4	0.89 (5)	2.84 (5)	3.677 (4)	158 (4)
N5—H5*B*⋯S4^i^	0.93 (5)	2.70 (5)	3.613 (3)	167 (4)
N7—H7*C*⋯S2	0.89 (5)	2.86 (4)	3.542 (4)	135 (3)
N7—H7*C*⋯S3	0.89 (5)	2.80 (5)	3.656 (4)	162 (4)
N10—H10*B*⋯S6	0.89 (6)	2.84 (6)	3.718 (4)	169 (4)
N10—H10*A*⋯S6^ii^	0.93 (5)	2.65 (5)	3.545 (4)	163 (3)
N14—H14*C*⋯S5	0.93 (5)	2.90 (5)	3.552 (4)	128 (4)
N14—H14*C*⋯S6	0.93 (5)	2.86 (5)	3.781 (4)	170 (4)
N1—H1*C*⋯S3^iii^	0.88 (5)	2.98 (5)	3.782 (4)	154 (4)
N1—H1*A*⋯S5^i^	0.90 (5)	2.87 (5)	3.741 (4)	165 (4)
N1—H1*B*⋯S6^iv^	0.83 (5)	2.80 (5)	3.632 (4)	179 (4)
N2—H2*B*⋯S1^v^	0.82 (5)	2.71 (5)	3.521 (4)	168 (4)
N2—H2*A*⋯S5^iv^	0.83 (5)	2.76 (5)	3.566 (4)	166 (4)
N2—H2*C*⋯S6^ii^	0.91 (5)	2.66 (5)	3.569 (4)	171 (4)
N3—H3*C*⋯S1^v^	0.85 (6)	2.84 (6)	3.665 (4)	163 (4)
N3—H3*B*⋯S3^vi^	0.92 (5)	2.92 (5)	3.813 (4)	164 (4)
N4—H4*A*⋯S6^ii^	0.90 (6)	2.95 (6)	3.834 (4)	168 (4)
N5—H5*B*⋯S5^i^	0.93 (5)	2.91 (5)	3.587 (4)	130 (3)
N6—H6*C*⋯S5^iv^	0.81 (5)	2.91 (6)	3.663 (4)	156 (4)
N6—H6*B*⋯S6^ii^	0.90 (5)	2.78 (5)	3.664 (4)	169 (3)
N7—H7*A*⋯S4	0.83 (5)	2.89 (5)	3.691 (4)	161 (4)
N7—H7*B*⋯S6^ii^	0.84 (6)	2.85 (6)	3.600 (4)	150 (5)
N8—H8*B*⋯S1^i^	0.92 (5)	2.83 (5)	3.734 (4)	169 (3)
N8—H8*C*⋯S2^vi^	0.77 (5)	2.89 (5)	3.641 (4)	168 (5)
N8—H8*A*⋯S4^i^	0.83 (5)	2.68 (6)	3.507 (4)	177 (5)
N9—H9*A*⋯S1^i^	0.87 (5)	2.96 (5)	3.803 (4)	163 (4)
N9—H9*C*⋯S3^ii^	0.88 (5)	2.85 (5)	3.729 (4)	179 (4)
N9—H9*B*⋯S6	0.86 (5)	2.99 (5)	3.805 (4)	157 (4)
N11—H11*B*⋯S3^vi^	0.83 (5)	2.90 (5)	3.717 (4)	170 (4)
N12—H12*A*⋯S2	0.86 (5)	3.01 (5)	3.842 (4)	166 (4)
N12—H12*C*⋯S3^vii^	0.91 (5)	2.93 (5)	3.787 (4)	157 (3)
N13—H13*B*⋯S1^viii^	0.86 (5)	2.72 (5)	3.571 (4)	168 (4)
N13—H13*A*⋯S3^ix^	0.85 (5)	3.01 (5)	3.855 (4)	175 (4)
N13—H13*C*⋯N4^i^	0.86 (5)	2.62 (5)	3.412 (6)	154 (4)
N14—H14*B*⋯S3^vii^	0.85 (5)	2.83 (5)	3.603 (4)	153 (4)

Symmetry codes: (i) 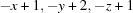; (ii) 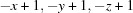; (iii) 
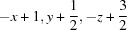
; (iv) 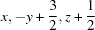; (v) 
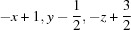
; (vi) 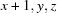; (vii) 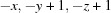; (viii) 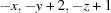; (ix) 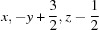.

**Fig. 1.**
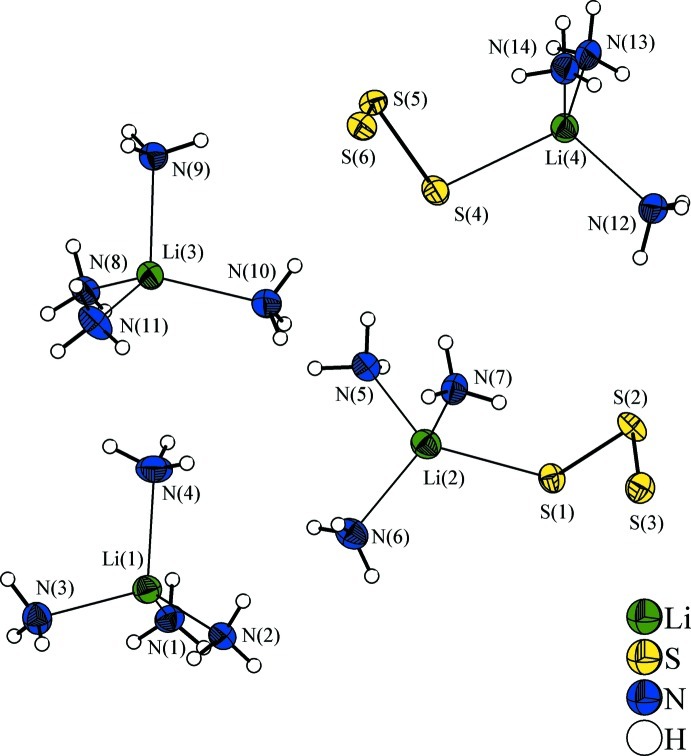
Asymmetric unit of [Li(NH3)4][Li(NH3)3S3]. Ellipsoids of all non-hydrogen atoms are given with a probability level of 50%.

**Fig. 2.**
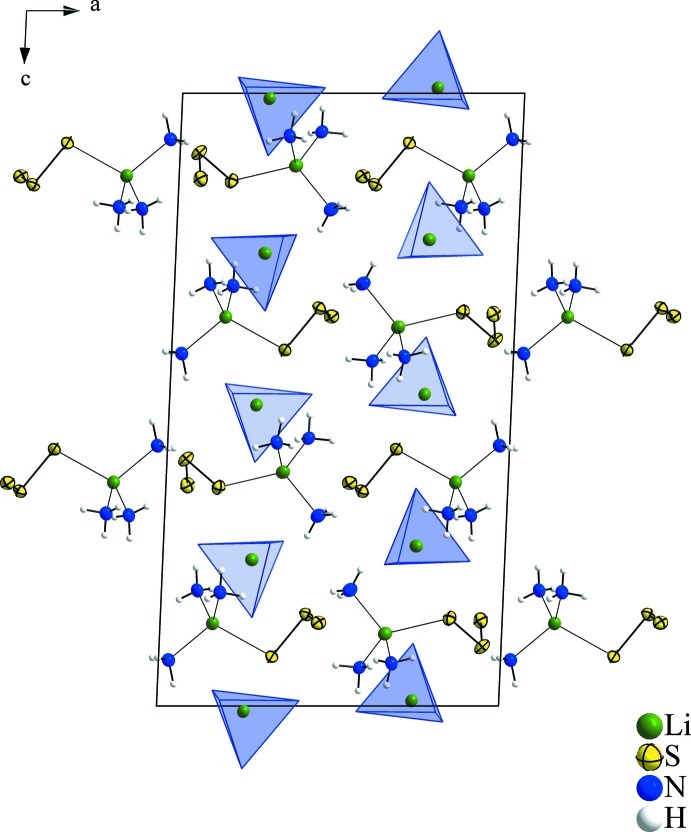
Projection of the unit cell of [Li(NH3)4][Li(NH3)3S3]. No hydrogen bondings are illustrated. Lithium tetraammine complexes are shown in blue polyhedron design without hydrogen atoms. The probability level of the displacement ellipsoids is 50%.

## supplementary crystallographic information

### Comment

In contrast to the trisulfides of the heavier alkali metals (Na–Cs) which were synthesized by Böttcher (1977, 1980*a*,*b*) under ammonothermalic conditions (130–400 °C, 500–3000 bar), [Li(NH_3_)_4_][Li(NH_3_)_3_S_3_] was formed in the reaction of lithium and sulfur in liquid ammonia. The crystal structure of [Li(NH_3_)_4_][Li(NH_3_)_3_S_3_] was determined in the course of investigations concerning the reactivity of sulfur containing components in solutions of alkali metals in liquid ammonia.

In the title compound, two crystallographically independent formula units represent the asymmetric unit (Fig. 1). The independent trisulfide anions S_3_^2–^ have an angled shape with angles of 110.43 (5)° and 109.53 (5)°. The average of the sulfur-sulfur distances is 2.083 Å and agrees with known S—S-distances of other trisulfides (Böttcher, 1977, 1980*a*,*b*). In contrast to the isolated Se_3_^2–^-anion in [Li(NH_3_)_4_]Se_3,_ the two crystallographically different S_3_^2–^-anions build mono anionic [Li(NH_3_)_3_S_3_]^-^-aggregates with triammine complexes. Therein, the lithium atoms are pseudo-tetrahedrally surrounded by three nitrogen and one sulfur atom. Mono cationic lithium tetrammine complexes compensate the remaining negative charges. In the [Li(NH_3_)_4_]^+^– and [Li(NH_3_)_3_S_3_]^-^-units the Li—N-distances range from 2.033 (7) Å to 2.105 (7) Å, the two Li—S-distances from 2.503 (5) Å to 2.547 (6) Å. The title compound represents after Na_2_S_3_ × NH_3_ the second trisulfide compound that contains ammonia molecules of crystallization. Every ammonia molecule forms hydrogen bonds and acts as a donor molecule. The sulfur atoms and the nitrogen atom N(4) operate as hydrogen bond acceptors. The proton···sulfur distance corresponds to similar hydrogen bonds in compounds synthesized by Rossmeier (2002, 2005) or Meier (2008). Distances and angles are shown in Table 1. Figure 2 illustrates the unit cell of the title compound but hydrogen bonds are not depicted.

### Experimental

All preparations were carried out in an atmosphere of dried and purified argon using standard Schlenk techniques. Liquid ammonia was dried and stored over sodium. 100 mg (14.2 mmol) Li and 231 mg (7.2 mmol) S_8_ were placed in a baked out U-Schlenk tube inside a glove box. Approximately 25 ml ammonia were condensed into the tube at -78°C, yielding a blue solution of the alkali metal. After a storage at -38°C for three weeks the solution colour turned to yellow-orange and after four months orange crystals were formed. One was subjected to low temperature X-ray diffraction.

### Refinement

All hydrogen atoms were found by difference Fourier analysis and refined isotropically.

### Crystal data

**Table:**

[Li(NH_3_)_4_][Li(NH_3_)_3_S_3_]	*F*(000) = 992
*M**_r_* = 229.30	*D*_x_ = 1.176 Mg m^−^^3^
Monoclinic, *P*2_1_/*c*	Mo *K*α radiation, λ = 0.71073 Å
Hall symbol: -P 2ybc	Cell parameters from 33516 reflections
*a* = 12.422 (3) Å	θ = 2.4–25.5°
*b* = 9.3721 (19) Å	µ = 0.54 mm^−^^1^
*c* = 22.269 (5) Å	*T* = 123 K
β = 92.46 (3)°	Block, orange
*V* = 2590.2 (9) Å^3^	0.1 × 0.1 × 0.1 mm
*Z* = 8	

### Data collection

**Table:**

Stoe IPDS 1 diffractometer	4788 independent reflections
Radiation source: fine-focus sealed tube	2989 reflections with *I* > 2σ(*I*)
Graphite monochromator	*R*_int_ = 0.097
rotation scans	θ_max_ = 25.5°, θ_min_ = 2.4°
Absorption correction: numerical (*X-SHAPE* and *X-RED*; Stoe & Cie 2006)	*h* = −15→15
*T*_min_ = 0.947, *T*_max_ = 0.981	*k* = −11→11
32304 measured reflections	*l* = −26→26

### Refinement

**Table:**

Refinement on *F*^2^	Primary atom site location: structure-invariant direct methods
Least-squares matrix: full	Secondary atom site location: difference Fourier map
*R*[*F*^2^ > 2σ(*F*^2^)] = 0.040	Hydrogen site location: difference Fourier map
*wR*(*F*^2^) = 0.079	All H-atom parameters refined
*S* = 0.81	*w* = 1/[σ^2^(*F*_o_^2^) + (0.0278*P*)^2^] where *P* = (*F*_o_^2^ + 2*F*_c_^2^)/3
4788 reflections	(Δ/σ)_max_ = 0.001
383 parameters	Δρ_max_ = 0.53 e Å^−^^3^
0 restraints	Δρ_min_ = −0.26 e Å^−^^3^

### Special details

**Table:**

Experimental. Crystal mounting in perfluorether
Geometry. All e.s.d.'s (except the e.s.d. in the dihedral angle between two l.s. planes) are estimated using the full covariance matrix. The cell e.s.d.'s are taken into account individually in the estimation of e.s.d.'s in distances, angles and torsion angles; correlations between e.s.d.'s in cell parameters are only used when they are defined by crystal symmetry. An approximate (isotropic) treatment of cell e.s.d.'s is used for estimating e.s.d.'s involving l.s. planes.
Refinement. Refinement of *F*^2^ against ALL reflections. The weighted *R*-factor *wR* and goodness of fit *S* are based on *F*^2^, conventional *R*-factors *R* are based on *F*, with *F* set to zero for negative *F*^2^. The threshold expression of *F*^2^ > σ(*F*^2^) is used only for calculating *R*-factors(gt) *etc*. and is not relevant to the choice of reflections for refinement. *R*-factors based on *F*^2^ are statistically about twice as large as those based on *F*, and *R*- factors based on ALL data will be even larger.

### Fractional atomic coordinates and isotropic or equivalent isotropic displacement parameters (Å^2^)

**Table:**

	*x*	*y*	*z*	*U*_iso_*/*U*_eq_	
Li1	0.7395 (4)	0.7907 (6)	0.7389 (3)	0.0252 (13)	
Li2	0.3406 (4)	0.8493 (7)	0.6182 (3)	0.0266 (13)	
Li3	0.7452 (4)	0.7081 (6)	0.4913 (3)	0.0250 (13)	
Li4	0.1536 (4)	0.7795 (6)	0.3651 (3)	0.0249 (13)	
S1	0.15394 (6)	0.93809 (9)	0.64317 (4)	0.02269 (19)	
S2	0.05542 (7)	0.79073 (9)	0.59691 (4)	0.0255 (2)	
S3	0.06305 (7)	0.59281 (9)	0.63965 (4)	0.0269 (2)	
S4	0.33097 (7)	0.82682 (10)	0.41901 (4)	0.0268 (2)	
S5	0.42615 (6)	0.75535 (9)	0.35064 (4)	0.02202 (18)	
S6	0.46313 (7)	0.54071 (10)	0.36418 (4)	0.0240 (2)	
N1	0.6879 (3)	0.9930 (4)	0.76601 (16)	0.0265 (7)	
N2	0.6495 (3)	0.6230 (4)	0.76905 (16)	0.0244 (7)	
N3	0.8984 (3)	0.7513 (5)	0.76394 (17)	0.0288 (7)	
N4	0.7316 (3)	0.7768 (5)	0.64448 (15)	0.0358 (8)	
N5	0.4113 (3)	0.9960 (4)	0.56295 (15)	0.0246 (7)	
N6	0.4488 (3)	0.8081 (4)	0.68949 (17)	0.0294 (7)	
N7	0.3186 (3)	0.6668 (4)	0.56994 (17)	0.0266 (7)	
N8	0.8168 (3)	0.9077 (4)	0.51002 (17)	0.0246 (7)	
N9	0.7427 (3)	0.6940 (4)	0.39786 (14)	0.0257 (7)	
N10	0.5838 (2)	0.6757 (4)	0.50895 (16)	0.0275 (7)	
N11	0.8388 (3)	0.5406 (4)	0.52589 (18)	0.0326 (8)	
N12	0.0296 (2)	0.7627 (4)	0.42483 (15)	0.0270 (7)	
N13	0.1046 (3)	0.9471 (4)	0.31136 (17)	0.0266 (7)	
N14	0.1714 (3)	0.5983 (4)	0.31432 (18)	0.0292 (7)	
H1A	0.678 (3)	1.056 (5)	0.737 (2)	0.038 (12)*	
H1B	0.640 (3)	0.989 (4)	0.7876 (19)	0.025 (11)*	
H1C	0.733 (3)	1.046 (5)	0.7866 (19)	0.032 (11)*	
H2A	0.603 (3)	0.637 (4)	0.7916 (19)	0.026 (8)*	
H2B	0.695 (4)	0.563 (5)	0.786 (2)	0.040 (12)*	
H2C	0.625 (3)	0.573 (5)	0.740 (2)	0.035 (12)*	
H3A	0.925 (4)	0.810 (6)	0.780 (2)	0.041 (15)*	
H3B	0.942 (3)	0.732 (5)	0.7338 (19)	0.033 (11)*	
H3C	0.899 (4)	0.674 (6)	0.782 (2)	0.046 (14)*	
H4A	0.693 (4)	0.699 (6)	0.637 (2)	0.055 (15)*	
H4B	0.795 (4)	0.769 (6)	0.628 (2)	0.058 (15)*	
H4C	0.706 (4)	0.845 (5)	0.625 (2)	0.039 (9)*	
H5A	0.412 (3)	0.967 (5)	0.525 (2)	0.034 (11)*	
H5B	0.481 (4)	1.025 (4)	0.5667 (18)	0.033 (11)*	
H5C	0.380 (3)	1.074 (5)	0.5647 (19)	0.033 (12)*	
H6A	0.497 (4)	0.863 (5)	0.6871 (19)	0.035 (13)*	
H6B	0.476 (3)	0.725 (5)	0.6808 (17)	0.021 (10)*	
H6C	0.420 (4)	0.809 (5)	0.722 (2)	0.045 (14)*	
H7A	0.330 (3)	0.680 (5)	0.534 (2)	0.034 (12)*	
H7B	0.362 (4)	0.593 (6)	0.575 (2)	0.055 (15)*	
H7C	0.258 (4)	0.640 (4)	0.5784 (18)	0.030 (11)*	
H8A	0.784 (4)	0.967 (5)	0.527 (2)	0.039 (9)*	
H8B	0.830 (3)	0.953 (4)	0.477 (2)	0.025 (10)*	
H8C	0.871 (4)	0.895 (5)	0.529 (2)	0.035 (12)*	
H9A	0.770 (3)	0.767 (5)	0.3810 (19)	0.035 (12)*	
H9B	0.677 (4)	0.683 (5)	0.3837 (19)	0.039 (12)*	
H9C	0.785 (4)	0.628 (5)	0.389 (2)	0.041 (13)*	
H10A	0.571 (3)	0.603 (4)	0.5340 (18)	0.022 (10)*	
H10B	0.547 (4)	0.647 (5)	0.478 (2)	0.048 (14)*	
H10C	0.552 (3)	0.744 (5)	0.526 (2)	0.041 (13)*	
H11A	0.806 (3)	0.481 (5)	0.5431 (19)	0.025 (12)*	
H11B	0.889 (4)	0.563 (5)	0.550 (2)	0.039 (12)*	
H11C	0.870 (3)	0.496 (5)	0.497 (2)	0.031 (12)*	
H12A	0.047 (3)	0.767 (5)	0.464 (2)	0.041 (12)*	
H12B	−0.018 (4)	0.824 (6)	0.421 (2)	0.053 (15)*	
H12C	−0.002 (3)	0.678 (5)	0.4214 (18)	0.029 (11)*	
H13A	0.099 (3)	0.931 (4)	0.274 (2)	0.026 (11)*	
H13B	0.053 (4)	0.978 (5)	0.326 (2)	0.036 (13)*	
H13C	0.146 (3)	1.020 (5)	0.3085 (17)	0.026 (8)*	
H14A	0.173 (4)	0.610 (5)	0.279 (3)	0.047 (15)*	
H14B	0.136 (3)	0.533 (5)	0.3247 (19)	0.030 (12)*	
H14C	0.238 (4)	0.574 (5)	0.321 (2)	0.050 (13)*	

### Atomic displacement parameters (Å^2^)

**Table:**

	*U*^11^	*U*^22^	*U*^33^	*U*^12^	*U*^13^	*U*^23^
Li1	0.025 (3)	0.027 (3)	0.024 (3)	0.003 (2)	0.001 (2)	0.001 (3)
Li2	0.028 (3)	0.026 (3)	0.026 (3)	0.001 (2)	−0.001 (2)	−0.003 (3)
Li3	0.022 (3)	0.029 (4)	0.024 (3)	−0.001 (2)	−0.001 (2)	0.003 (3)
Li4	0.023 (3)	0.026 (3)	0.026 (3)	0.001 (2)	0.000 (2)	0.003 (3)
S1	0.0216 (4)	0.0202 (4)	0.0260 (5)	−0.0005 (3)	−0.0009 (3)	−0.0036 (4)
S2	0.0259 (4)	0.0251 (5)	0.0245 (4)	−0.0007 (3)	−0.0098 (3)	−0.0003 (4)
S3	0.0277 (5)	0.0234 (5)	0.0297 (5)	−0.0051 (3)	0.0010 (4)	0.0013 (4)
S4	0.0214 (4)	0.0338 (5)	0.0250 (5)	0.0051 (4)	−0.0018 (3)	−0.0094 (4)
S5	0.0242 (4)	0.0222 (4)	0.0198 (4)	0.0007 (3)	0.0031 (3)	0.0017 (4)
S6	0.0265 (4)	0.0215 (5)	0.0238 (4)	0.0027 (3)	−0.0008 (3)	−0.0024 (4)
N1	0.0265 (17)	0.0296 (18)	0.0234 (17)	0.0007 (14)	0.0031 (15)	0.0043 (15)
N2	0.0226 (16)	0.0283 (18)	0.0221 (17)	0.0011 (13)	−0.0011 (13)	0.0007 (14)
N3	0.0259 (16)	0.0283 (19)	0.0321 (19)	0.0006 (16)	−0.0011 (14)	0.0035 (18)
N4	0.044 (2)	0.041 (2)	0.0219 (17)	−0.0086 (18)	−0.0003 (15)	0.0037 (16)
N5	0.0241 (17)	0.0241 (18)	0.0254 (18)	−0.0003 (13)	−0.0021 (13)	0.0003 (14)
N6	0.0314 (18)	0.0277 (19)	0.0285 (19)	0.0048 (17)	−0.0032 (15)	−0.0018 (16)
N7	0.0223 (16)	0.0275 (19)	0.030 (2)	−0.0030 (14)	0.0037 (14)	−0.0014 (15)
N8	0.0241 (17)	0.0252 (17)	0.0242 (18)	0.0029 (13)	−0.0022 (14)	−0.0017 (15)
N9	0.0257 (17)	0.0278 (18)	0.0235 (16)	−0.0027 (15)	0.0002 (13)	0.0016 (14)
N10	0.0271 (16)	0.0304 (19)	0.0249 (18)	−0.0013 (15)	0.0001 (14)	0.0032 (16)
N11	0.0355 (18)	0.0266 (19)	0.034 (2)	−0.0016 (16)	−0.0142 (17)	0.0017 (17)
N12	0.0250 (15)	0.0290 (19)	0.0271 (18)	0.0021 (15)	0.0022 (13)	0.0000 (15)
N13	0.0225 (16)	0.0289 (19)	0.0287 (19)	−0.0054 (14)	0.0040 (14)	0.0032 (15)
N14	0.0244 (18)	0.034 (2)	0.029 (2)	−0.0008 (16)	0.0024 (14)	−0.0054 (16)

### Geometric parameters (Å, º)

**Table:**

Li1—N2	2.059 (7)	N4—H4C	0.83 (5)
Li1—N3	2.061 (6)	N5—H5A	0.88 (5)
Li1—N1	2.099 (7)	N5—H5B	0.90 (4)
Li1—N4	2.104 (7)	N5—H5C	0.83 (5)
Li2—N7	2.033 (7)	N6—H6A	0.80 (5)
Li2—N5	2.066 (7)	N6—H6B	0.87 (4)
Li2—N6	2.071 (7)	N6—H6C	0.82 (5)
Li2—S1	2.547 (6)	N7—H7A	0.82 (5)
Li3—N10	2.081 (6)	N7—H7B	0.88 (6)
Li3—N11	2.082 (7)	N7—H7C	0.83 (4)
Li3—N9	2.085 (7)	N8—H8A	0.80 (5)
Li3—N8	2.105 (7)	N8—H8B	0.86 (4)
Li4—N13	2.052 (7)	N8—H8C	0.79 (5)
Li4—N14	2.058 (7)	N9—H9A	0.86 (5)
Li4—N12	2.084 (7)	N9—H9B	0.87 (5)
Li4—S4	2.503 (5)	N9—H9C	0.84 (5)
S1—S2	2.0876 (13)	N10—H10A	0.90 (4)
S2—S3	2.0852 (13)	N10—H10B	0.85 (5)
S4—S5	2.0782 (13)	N10—H10C	0.85 (5)
S5—S6	2.0825 (13)	N11—H11A	0.80 (4)
N1—H1A	0.87 (5)	N11—H11B	0.83 (5)
N1—H1B	0.79 (4)	N11—H11C	0.87 (5)
N1—H1C	0.86 (5)	N12—H12A	0.89 (5)
N2—H2A	0.79 (4)	N12—H12B	0.83 (5)
N2—H2B	0.87 (5)	N12—H12C	0.89 (5)
N2—H2C	0.85 (5)	N13—H13A	0.84 (4)
N3—H3A	0.73 (5)	N13—H13B	0.78 (5)
N3—H3B	0.90 (4)	N13—H13C	0.86 (4)
N3—H3C	0.83 (5)	N14—H14A	0.80 (5)
N4—H4A	0.88 (6)	N14—H14B	0.79 (5)
N4—H4B	0.89 (5)	N14—H14C	0.87 (5)
			
N2—Li1—N3	107.6 (3)	H5A—N5—H5C	110 (4)
N2—Li1—N1	114.8 (3)	H5B—N5—H5C	100 (4)
N3—Li1—N1	112.6 (3)	Li2—N6—H6A	107 (3)
N2—Li1—N4	106.0 (3)	Li2—N6—H6B	104 (2)
N3—Li1—N4	105.3 (3)	H6A—N6—H6B	105 (4)
N1—Li1—N4	110.0 (3)	Li2—N6—H6C	112 (3)
N7—Li2—N5	107.2 (3)	H6A—N6—H6C	115 (5)
N7—Li2—N6	108.5 (3)	H6B—N6—H6C	113 (4)
N5—Li2—N6	107.6 (3)	Li2—N7—H7A	111 (3)
N7—Li2—S1	106.7 (3)	Li2—N7—H7B	122 (3)
N5—Li2—S1	109.1 (3)	H7A—N7—H7B	96 (4)
N6—Li2—S1	117.4 (3)	Li2—N7—H7C	104 (3)
N10—Li3—N11	110.3 (3)	H7A—N7—H7C	118 (4)
N10—Li3—N9	101.9 (3)	H7B—N7—H7C	107 (4)
N11—Li3—N9	107.8 (3)	Li3—N8—H8A	120 (3)
N10—Li3—N8	119.7 (3)	Li3—N8—H8B	111 (3)
N11—Li3—N8	111.9 (3)	H8A—N8—H8B	100 (4)
N9—Li3—N8	104.0 (3)	Li3—N8—H8C	108 (3)
N13—Li4—N14	110.3 (3)	H8A—N8—H8C	107 (4)
N13—Li4—N12	102.8 (3)	H8B—N8—H8C	109 (4)
N14—Li4—N12	112.8 (3)	Li3—N9—H9A	113 (3)
N13—Li4—S4	112.2 (3)	Li3—N9—H9B	110 (3)
N14—Li4—S4	107.3 (2)	H9A—N9—H9B	109 (4)
N12—Li4—S4	111.5 (3)	Li3—N9—H9C	108 (3)
S2—S1—Li2	101.31 (14)	H9A—N9—H9C	103 (4)
S3—S2—S1	110.43 (5)	H9B—N9—H9C	114 (4)
S5—S4—Li4	96.22 (14)	Li3—N10—H10A	116 (2)
S4—S5—S6	109.53 (5)	Li3—N10—H10B	112 (3)
Li1—N1—H1A	116 (3)	H10A—N10—H10B	99 (4)
Li1—N1—H1B	113 (3)	Li3—N10—H10C	116 (3)
H1A—N1—H1B	113 (4)	H10A—N10—H10C	101 (4)
Li1—N1—H1C	118 (3)	H10B—N10—H10C	111 (4)
H1A—N1—H1C	94 (4)	Li3—N11—H11A	115 (3)
H1B—N1—H1C	101 (4)	Li3—N11—H11B	116 (3)
Li1—N2—H2A	120 (3)	H11A—N11—H11B	105 (4)
Li1—N2—H2B	106 (3)	Li3—N11—H11C	110 (3)
H2A—N2—H2B	108 (4)	H11A—N11—H11C	106 (4)
Li1—N2—H2C	111 (3)	H11B—N11—H11C	105 (4)
H2A—N2—H2C	109 (4)	Li4—N12—H12A	118 (3)
H2B—N2—H2C	101 (4)	Li4—N12—H12B	116 (3)
Li1—N3—H3A	114 (4)	H12A—N12—H12B	101 (4)
Li1—N3—H3B	116 (2)	Li4—N12—H12C	111 (3)
H3A—N3—H3B	104 (4)	H12A—N12—H12C	102 (4)
Li1—N3—H3C	106 (3)	H12B—N12—H12C	107 (4)
H3A—N3—H3C	114 (5)	Li4—N13—H13A	117 (3)
H3B—N3—H3C	102 (4)	Li4—N13—H13B	106 (3)
Li1—N4—H4A	103 (3)	H13A—N13—H13B	115 (4)
Li1—N4—H4B	115 (3)	Li4—N13—H13C	119 (3)
H4A—N4—H4B	110 (5)	H13A—N13—H13C	95 (4)
Li1—N4—H4C	118 (3)	H13B—N13—H13C	104 (4)
H4A—N4—H4C	110 (4)	Li4—N14—H14A	116 (4)
H4B—N4—H4C	100 (4)	Li4—N14—H14B	114 (3)
Li2—N5—H5A	113 (3)	H14A—N14—H14B	116 (5)
Li2—N5—H5B	125 (3)	Li4—N14—H14C	104 (3)
H5A—N5—H5B	98 (4)	H14A—N14—H14C	99 (4)
Li2—N5—H5C	110 (3)	H14B—N14—H14C	106 (4)

### Hydrogen-bond geometry (Å, º)

**Table:**

*D*—H···*A*	*D*—H	H···*A*	*D*···*A*	*D*—H···*A*
N5—H5*A*···S4	0.89 (5)	2.84 (5)	3.677 (4)	158 (4)
N5—H5*B*···S4^i^	0.93 (5)	2.70 (5)	3.613 (3)	167 (4)
N7—H7*C*···S2	0.89 (5)	2.86 (4)	3.542 (4)	135 (3)
N7—H7*C*···S3	0.89 (5)	2.80 (5)	3.656 (4)	162 (4)
N10—H10*B*···S6	0.89 (6)	2.84 (6)	3.718 (4)	169 (4)
N10—H10*A*···S6^ii^	0.93 (5)	2.65 (5)	3.545 (4)	163 (3)
N14—H14*C*···S5	0.93 (5)	2.90 (5)	3.552 (4)	128 (4)
N14—H14*C*···S6	0.93 (5)	2.86 (5)	3.781 (4)	170 (4)
N1—H1*C*···S3^iii^	0.88 (5)	2.98 (5)	3.782 (4)	154 (4)
N1—H1*A*···S5^i^	0.90 (5)	2.87 (5)	3.741 (4)	165 (4)
N1—H1*B*···S6^iv^	0.83 (5)	2.80 (5)	3.632 (4)	179 (4)
N2—H2*B*···S1^v^	0.82 (5)	2.71 (5)	3.521 (4)	168 (4)
N2—H2*A*···S5^iv^	0.83 (5)	2.76 (5)	3.566 (4)	166 (4)
N2—H2*C*···S6^ii^	0.91 (5)	2.66 (5)	3.569 (4)	171 (4)
N3—H3*C*···S1^v^	0.85 (6)	2.84 (6)	3.665 (4)	163 (4)
N3—H3*B*···S3^vi^	0.92 (5)	2.92 (5)	3.813 (4)	164 (4)
N4—H4*A*···S6^ii^	0.90 (6)	2.95 (6)	3.834 (4)	168 (4)
N5—H5*B*···S5^i^	0.93 (5)	2.91 (5)	3.587 (4)	130 (3)
N6—H6*C*···S5^iv^	0.81 (5)	2.91 (6)	3.663 (4)	156 (4)
N6—H6*B*···S6^ii^	0.90 (5)	2.78 (5)	3.664 (4)	169 (3)
N7—H7*A*···S4	0.83 (5)	2.89 (5)	3.691 (4)	161 (4)
N7—H7*B*···S6^ii^	0.84 (6)	2.85 (6)	3.600 (4)	150 (5)
N8—H8*B*···S1^i^	0.92 (5)	2.83 (5)	3.734 (4)	169 (3)
N8—H8*C*···S2^vi^	0.77 (5)	2.89 (5)	3.641 (4)	168 (5)
N8—H8*A*···S4^i^	0.83 (5)	2.68 (6)	3.507 (4)	177 (5)
N9—H9*A*···S1^i^	0.87 (5)	2.96 (5)	3.803 (4)	163 (4)
N9—H9*C*···S3^ii^	0.88 (5)	2.85 (5)	3.729 (4)	179 (4)
N9—H9*B*···S6	0.86 (5)	2.99 (5)	3.805 (4)	157 (4)
N11—H11*B*···S3^vi^	0.83 (5)	2.90 (5)	3.717 (4)	170 (4)
N12—H12*A*···S2	0.86 (5)	3.01 (5)	3.842 (4)	166 (4)
N12—H12*C*···S3^vii^	0.91 (5)	2.93 (5)	3.787 (4)	157 (3)
N13—H13*B*···S1^viii^	0.86 (5)	2.72 (5)	3.571 (4)	168 (4)
N13—H13*A*···S3^ix^	0.85 (5)	3.01 (5)	3.855 (4)	175 (4)
N13—H13*C*···N4^i^	0.86 (5)	2.62 (5)	3.412 (6)	154 (4)
N14—H14*B*···S3^vii^	0.85 (5)	2.83 (5)	3.603 (4)	153 (4)

Symmetry codes: (i) −*x*+1, −*y*+2, −*z*+1; (ii) −*x*+1, −*y*+1, −*z*+1; (iii) −*x*+1, *y*+1/2, −*z*+3/2; (iv) *x*, −*y*+3/2, *z*+1/2; (v) −*x*+1, *y*−1/2, −*z*+3/2; (vi) *x*+1, *y*, *z*; (vii) −*x*, −*y*+1, −*z*+1; (viii) −*x*, −*y*+2, −*z*+1; (ix) *x*, −*y*+3/2, *z*−1/2.
